# A clinical combined gadobutrol bolus and slow infusion protocol enabling angiography, inversion recovery whole heart, and late gadolinium enhancement imaging in a single study

**DOI:** 10.1186/s12968-016-0285-7

**Published:** 2016-10-05

**Authors:** Animesh Tandon, Lorraine James, Markus Henningsson, René M. Botnar, Amanda Potersnak, Gerald F. Greil, Tarique Hussain

**Affiliations:** 1 Department of Pediatrics, University of Texas Southwestern Medical Center, 5323 Harry Hines Blvd, Dallas, 75390 Texas USA; 2 Department of Radiology, University of Texas Southwestern Medical Center, 5323 Harry Hines Blvd, Dallas, 75390 Texas USA; 3 Pediatric Cardiology, Children’s Medical Center Dallas, 1935 Medical District Dr, Dallas, 75235 Texas USA; 4 Department of Imaging and Biomedical Engineering, King’s College London, London, UK; 5 Pontificia Universidad Católica de Chile, Escuela de Ingeniería, Santiago, Chile

**Keywords:** Congenital heart disease, Gadobutrol, Time-resolved magnetic resonance angiography, Steady state magnetic resonance angiography, Late gadolinium enhancement

## Abstract

**Background:**

The use of gadolinium contrast agents in cardiovascular magnetic resonance is well-established and serves to improve both vascular imaging as well as enable late gadolinium enhancement (LGE) imaging for tissue characterization. Currently, gadofosveset trisodium, an intravascular contrast agent, combined with a three-dimensional inversion recovery balanced steady state free precession (3D IR bSSFP) sequence, is commonly used in pediatric cardiac imaging and yields excellent vascular imaging, but cannot be used for late gadolinium enhancement. Gadofosveset use remains limited in clinical practice, and manufacture was recently halted, thus an alternative is needed to allow 3D IR bSSFP and LGE in the same study.

**Methods:**

Here we propose a protocol to give a bolus of 0.1 mL/kg = 0.1 mmol/kg gadobutrol (GADAVIST/GADOVIST) for time-resolved magnetic resonance angiography (MRA). Subsequently, 0.1 mmol/kg is diluted up to 5 or 7.5 mL with saline and then loaded into intravenous tubing connected to the patient. A 0.5 mL short bolus is infused, then a slow infusion is given at 0.02 or 0.03 mL/s. Image navigated (iNAV) 3D IR bSSFP imaging is initiated 45–60 s after the initiation of the infusion, with a total image acquisition time of ~5 min. If necessary, LGE imaging using phase sensitive inversion recovery reconstruction (PSIR) is performed at 10 min after the infusion is initiated.

**Results:**

We have successfully performed the above protocol with good image quality on 10 patients with both time-resolved MRA and 3D IR bSSFP iNAV imaging. Our initial attempts to use pencil beam respiratory navigation failed due to signal labeling in the liver by the navigator. We have also performed 2D PSIR LGE successfully, with both LGE positive and LGE negative results.

**Conclusion:**

A bolus of gadobutrol, followed later by a slow infusion, allows time-resolved MRA, 3D IR bSSFP using the iNAV navigation technique, and LGE imaging, all in a single study with a single contrast agent.

## Background

Delineation of vascular structures is an especially important part of cardiovascular magnetic resonance (CMR) in congenital heart disease (CHD), due to the frequency of vascular lesions. Current techniques for vascular imaging in CHD are in three major groups:

- Non-contrast three-dimensional (3D), ECG and respiratory navigated, T2-prepared, fat saturated imaging with a balanced steady-state free precession readout (3D T2-prep bSSFP).
- Contrast-enhanced non-gated conventional or time-resolved magnetic resonance angiography (MRA).
- And intravascular contrast-enhanced, 3D, ECG and respiratory navigated, fat saturated imaging with an inversion recovery pulse and balanced steady-state free precession readout (3D IR bSSFP) [1, 2].

This latter technique has been shown to be superior to non-contrast 3D T2-prep bSSFP with both intravascular and extravascular contrast agents [2], and to IR gradient recovery echo sequences [3]. The use of image navigation (iNAV) as opposed to pencil beam navigation further improves the image quality [4]. Thus, the standard of care for imaging at our center was gadofosveset trisodium (ABLAVAR, Lantheus Medical Imaging, N. Billerica, MA)-enhanced 3D IR bSSFP imaging. However, due to gadofosveset trisodium’s albumin binding, the kinetics are such that they preclude late gadolinium enhancement (LGE) imaging in the same study.

Gadofosveset is approved for use, but there is currently a disruption in manufacturing (August 1, 2016 (manufacturer communication)). Furthermore, clinical use of this contrast agent was not ubiquitous. Gadobenate dimeglumine (MultiHance, Bracco Diagnostic, Milano, Italy) has been shown to produce similar images as gadofosveset [5] given its partial albumin binding characteristics, but given its linear nature, there are theoretical concerns regarding a higher risk of central nervous system deposition [6] and nephrogenic systemic fibrosis (NSF) than with macrocyclic gadolinium compounds [7]. Gadobutrol (GADAVIST (Bayer Healthcare Pharmaceuticals, Whippany, NJ) or GADOVIST (Bayer Schering Pharma, Berlin, Germany)) is a macrocyclic, ionic gadolinium-containing contrast agent with no known cases of NSF and no protein-binding characteristics [7], and is FDA-approved in adult and pediatric patients (including term neonates) to detect and visualize areas with disrupted blood brain barrier and/or abnormal vascularity of the central nervous system, and for use in magnetic resonance angiography (MRA) in adult and pediatric patients (including term neonates) to evaluate known or suspected supra-aortic or renal artery disease [8]. Gadobutrol has shown equal efficacy for LGE imaging as compared to Gd-DTPA [9].

To address the current disruption of gadofosveset manufacture, we devised a method of dosing gadobutrol in order to achieve high-quality 3D IR bSSFP images while also allowing LGE imaging. Our team was inspired by Dabir et al. (2012) [10], who performed multi-dynamic first-pass MRA followed by single-phase T1-weighted 3D inversion recovery imaging with ECG triggering and respiratory navigator gating. However, they used a 3 Tesla MR scanner and a SENSE acceleration factor of 4, while our protocol used a lower SENSE acceleration factor of 2 at 1.5 Tesla. The novelty of the current study was the use of image navigated respiratory motion correction that further accelerated 3D IR bSSFP image acquisition, since a user-defined scan efficiency can be chosen prior to the scan [11]. In addition, the injector set up of this protocol allows a slow infusion protocol using a commonly available injector, without the need for a separate infusion pump; the use of the injector pump set-up was inspired by Ishida et al. (2011) [12]. The practicality of this protocol makes it easily applied in everyday practice.

## Methods

### Study population

Consecutive patients underdoing contrast-enhanced CMR with a clinical indication for 3D whole-heart imaging at our institution had the protocol performed. We have IRB approval for retrospective use of clinically-obtained data, and there is a waiver of consent.

### Equipment setup

The CMR studies were performed on an Ingenia 1.5 Tesla scanner (Philips, Best, The Netherlands) with the 32-channel phased array digital receiver coil for signal reception. The contrast power injector was a Medrad Spectris Solaris EP (Bayer, Whippany, NJ). The intravenous (IV) tubing used was Mede× 450FL 20 in., APV = 2.4 mL (Smiths Medical ASD Inc., Dublin, OH).

**Fig. 1:**
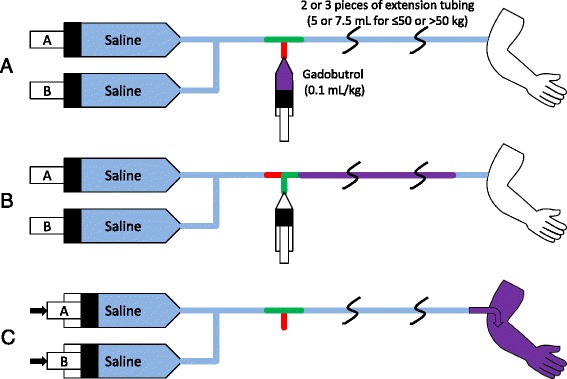
Gadobutrol bolus infusion setup. **a** Intravenous (IV) extension tubing is connected between the end of the injector line and the IV connected to the patient; either 2 pieces (if patient is ≤50 kg) or 3 pieces (if patient is >50 kg). This provides 5 or 7.5 mL of IV tubing volume between the injector and the patient, respectively. A three-way stopcock is attached between the injector and extension tubing. Both pumps of the power injector are loaded with saline. **b** When the patient is ready for time-resolved MRA, the technician turns the stopcock off to the injector, and 0.1 mL/kg undiluted gadobutrol is infused into the extension tubing. The power injector is set for pump A to inject 10 mL at 2 mL/s, and pump B is set to inject 20 mL/s at 2 mL/s. The stopcock is then turned back to allow flow from injector to patient. **c** Injection is then started at the appropriate time, and thus the contrast in the tubing is injected into the patient

**Fig. 2:**
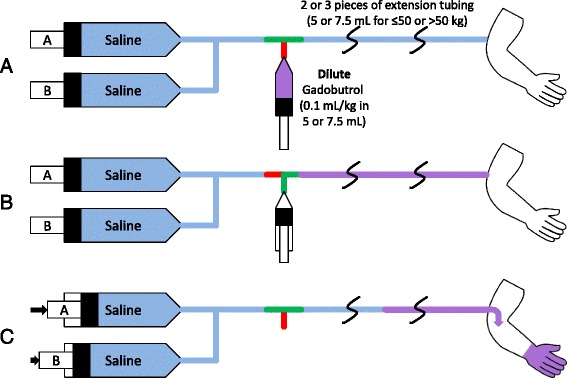
Gadobutrol slow infusion setup. **a** Gadobutrol 0.1 mL/kg is diluted with saline to 5 (≤50 kg patient) or 7.5 mL (>50 kg patient). **b** When ready for 3D IR bSSFP iNAV imaging, the dilute gadobutrol is loaded into the extension tubing. The 3D IR bSSFP iNAV sequence is set up to image for approximately 4 to 5 total minutes. **c** Injector pump A is set to inject 0.5 mL at 2 mL/s, to overcome dead space/saline in the extension tubing. Injector pump B is set to inject 20 mL at 0.02 mL/s (≤50 kg patient) or 0.03 mL/s (>50 kg patient). The injection is started, and after a delay of 45–60 s, 3D IR bSSFP iNAV imaging is started

An IV line is placed before the examination for contrast administration. Extension tubing is connected between the end of the injector line and the IV line connected to the patient (Figs. 1 and 2 explain the overall contrast and injector use; Fig. 1a defines extension tubing set-up); either 2 pieces (if patient was under 50 kg) or 3 pieces (if patient was over 50 kg). This provides ~5 or 7.5 mL of IV tubing volume between the injector and the patient. A three-way stopcock is attached between the injector and extension tubing. Both pumps of the power injector are loaded with saline.

### CMR protocol

#### Initial and gadobutrol bolus time-resolved MRA

**Fig. 3:**
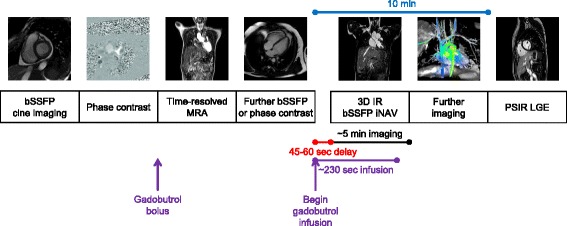
Example study timeline. This diagram graphically depicts the timeline of an example study using the gadobutrol bolus and slow infusion protocol

Standard bSSFP cine imaging is performed at the beginning of the study (Fig. 3), followed by phase contrast imaging. At the time of time-resolved MRA, undiluted gadobutrol is loaded into the tubing through the stopcock (Fig. 1b). At the appropriate time, the injection is started for time-resolved MRA. The gadobutrol in the extension tubing is thus injected into the patient. After time-resolved MRA, contrast-neutral imaging such as further phase contrast or bSSFP imaging is performed (Fig. 3).

#### Gadobutrol slow infusion

When the patient is ready for 3D IR bSSFP iNAV imaging, the patient is prepared for gadobutrol slow infusion, as shown in Fig. 2a. In short, diluted gadobutrol is loaded into the IV extension tubing through the stopcock. The 3D IR bSSFP iNAV sequence is set up to image for approximately 2 to 2.5 min with gating efficiency = 50 % (total imaging time 4 to 5 min). Field of view and voxel size are usually manipulated to get the correct imaging time; representative sequence parameters include repetition time/echo time (TR/TE) = 3.7/1.86 ms, flip angle = 70°, field-of-view (FOV) = 320 × 320 × 120 mm, voxel size = 1.5 × 1.5 × 1.5 mm, k space profile order was centric (low-high), SENSE factor = 2, inversion time = 220 ms (if run in systole) or 240 ms (if run in diastole) [4]. This sequence may be run in systole or diastole, depending on clinical indication, heart rate, and duration of the quiescent phases in the cardiac cycle [13].

When the 3D IR bSSFP iNAV is ready to run, the continuous infusion is started, and then after a 45–60 s delay, the imaging is started, as detailed in Figs. 2b, c, and 3. The pump rates suggested give about 225–234 s of injection of the contrast itself, followed by flush. The imaging sequence time extends beyond the time the contrast is infusing, but the initial delay between starting the injection and starting the imaging seems to allow the gadobutrol to build to a sufficient blood level.

#### Late gadolinium enhancement imaging

After the 3D IR bSSFP imaging is complete, further post-contrast imaging can be performed. At 10 to 15 min after the start of the infusion, 2D phase-sensitive inversion recovery (PSIR) LGE imaging can be performed, if necessary; representative sequence parameters include repetition time/echo time (TR/TE) = 5.5/6 ms, flip angle = 25°, echo train length = 22, in-plane resolution = 1.9 × 1.9 mm, slice thickness = 8 mm.

## Results

**Fig. 4:**
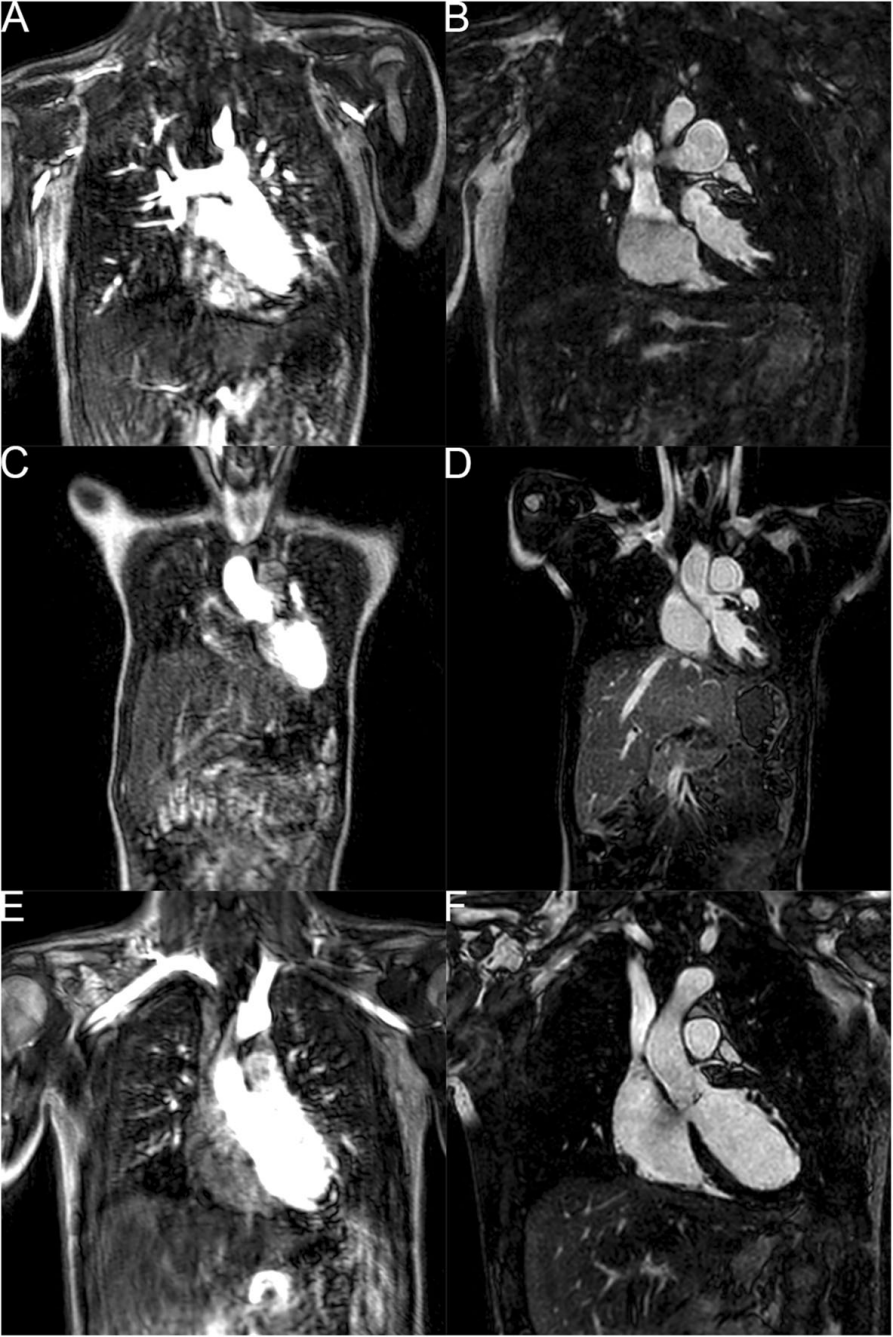
Time-resolved MRA and 3D IR bSSFP iNAV example images. MRA and 3D IR bSSFP iNAV images, respectively, from (**a**, **b**) patient with pulmonary stenosis and sinus venosus atrial septal defect (ASD) status post surgical intervention; (**c**, **d**) patient with hypoplastic right pulmonary artery (RPA) with severe distal RPA stenosis, secundum ASD, coronary sinus septal defect; (**e**, **f**) patient with bicuspid aortic valve with dilated aortic root

**Fig. 5:**
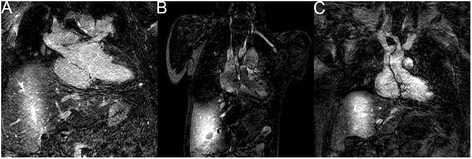
Example images of 3D IR bSSFP imaging using pencil beam navigation. As can be seen in all three panels, these proved to be unusable because the navigator labeled the blood in the liver and thus the overall image quality was degraded. This is perhaps due to the high signal in the liver causing an overall compression of the dynamic range. **a** Image from a patient with d-transposition of the great arteries, ventricular septal defect (VSD), and pulmonary stenosis status post aortic root translocation and VSD closure. **b** Image from a patient with a large secundum atrial septal defect. **c** Image from a patient with trisomy 21 and common AV canal status post surgical repair

**Fig. 6:**
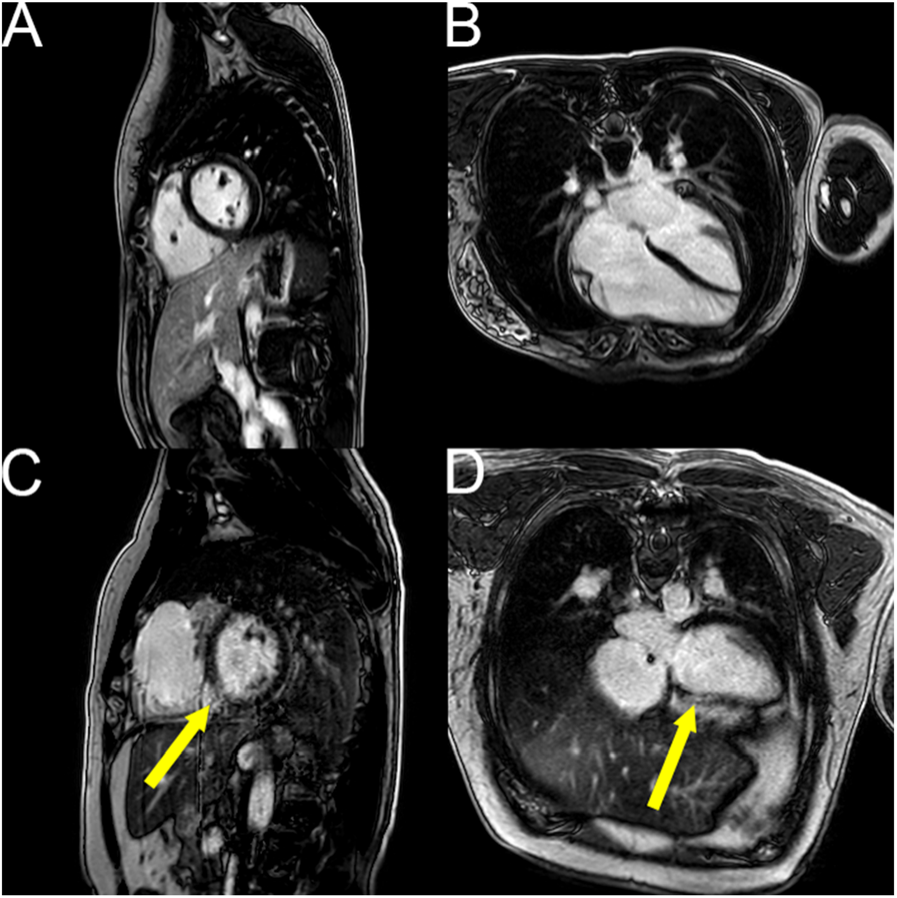
Example phase sensitive inversion recovery late gadolinium enhancement images. **a**, **b** LGE negative short axis (**a**) and 4 chamber (**b**) images from previously mentioned patient with pulmonary stenosis and sinus venosus atrial septal defect (ASD) status post surgical intervention. **c**, **d** A small area of full-thickness LGE (yellow arrows) in the mid inferoseptal segment correlating to the RCA distribution is shown in short axis (**a**) and 4 chamber (**b**) images from a patient with d-transposition of the great arteries, ventricular septal defect (VSD), and pulmonary stenosis status post aortic root translocation and VSD closure, and known previous right coronary kinking status post reimplantation

We successfully completed both time-resolved MRA and 3D IR bSSFP iNAV imaging with the above technique for 10 patients (Fig. 4) with a variety of diagnoses including: pulmonary stenosis and sinus venosus atrial septal defect (ASD) status post surgical intervention (Fig. 4a, b); hypoplastic right pulmonary artery (RPA) with severe distal RPA stenosis, secundum ASD, coronary sinus septal defect (Fig. 4c, d); and bicuspid aortic valve with dilated aortic root (Fig. 4e, f). The patient age ranged from 4.7 to 29.4 years (median 11.7 years); weight ranged from 15.1 to 92.4 kg (median 29.6 kg); body surface area was 0.64 to 2.02 m^2^ (median 1.10 m^2^); heart rate was 60–101 beats per minute (median 79); and all patients had normal creatinine for their age (0.3–1.0 mg/dL). Our initial three attempts used the pencil beam respiratory navigator with the 3D IR bSSFP sequence; however, all of these proved to be unusable. This is likely due to artifact generated by the navigator restore pulse labeling blood as has been described previously [14]. This likely caused a compression of the dynamic range and thus the overall image quality was degraded (Fig. 5). We have also run 2D PSIR LGE sequences as described above, with both positive and negative results (Fig. 6). Image quality for LGE was excellent in these cases.

## Conclusion

Given the importance of time-resolved MRA, steady state 3D IR bSSFP, and LGE in fully evaluating patients with congenital heart disease, our protocol represents a step forward in being able to acquire all of these datasets in a single exam with a single contrast agent. The individual features of this protocol are not novel. The slow infusion, image navigator, bolus technique and late gadolinium enhancement techniques have been described [10–12]. The purpose of the protocol is to provide practical guidance on how to combine these techniques to enable acquisition of these datasets using existing injector equipment at 1.5 Tesla magnet field strength. Our choice of inversion time was influenced by our previous clinical experience, and by previous work suggesting 280 ms for gadobutrol by Dabir et al. (2012) [10]. However, unlike that study, our contrast agent could not be completely injected as a slow infusion, because a bolus injection is required for first-pass MRA. This, in turn, results in a broader variation of contrast agent concentration in the blood and so benefits from a shorter inversion time. In addition, our goal was not to completely null the myocardium, as might be desired for coronary imaging, but to have some myocardial visualization to allow better identification of the anatomy in patients with congenital heart disease. We additionally confirmed our initial choice of 240 ms by running a Look-Locker immediately after the infusion. We used 220 ms for systole due to the practical need for a shorter inversion time at higher heart rates to allow systolic imaging. This protocol should be further evaluated in prospective studies to ensure that the image quality of all three image types are comparable to current standards, and to further optimize imaging parameters such as inversion time, image acquisition length compared to infusion duration, and other factors, but our subjective experience is that it delivers comparable images to gadofosveset trisodium-based 3D IR bSSFP while allowing the added advantage of LGE imaging. One potential alternative for centers that do not have access to image-based navigation techniques may be to use pencil beam navigation with a higher flip angle and the navigator restore pulse turned off. However, given that this reduces the effectiveness of navigation on the Philips platform, we did not evaluate this option. In addition, pencil-beam navigation with 3D IR GRE sequences on Siemens uses a different implementation of pulse sequences that does not require the navigator restore pulse. We would surmise, therefore, that these scanners can continue to use the conventional pencil-beam navigator without incurring the labeling artifact described above. Our correspondence with pediatric centers employing Siemens hardware supports this assumption (T. Slesnick, personal communication). Given the current disruption in gadofosveset trisodium production, the importance of this alternative protocol will be evident to CMR practitioners who care for patients with congenital heart disease.

## Abbreviations

3D IR bSSFP: Three-dimensional inversion recovery balanced steady state free precession
ASD: Atrial septal defect
iNAV: Image navigation
IV: Intravenous
LGE: Late gadolinium enhancement
MRA: Magnetic resonance angiography
NSF: Nephrogenic systemic fibrosis
PSIR: Phase-sensitive inversion recovery
RPA: Right pulmonary artery
